# SKNY-1, a THCV Analog, Produces Weight Loss, Lipid Normalization and Attenuation of Reward-Associated Behaviors in an mc4r(G894C) Zebrafish Model of Obesity

**DOI:** 10.3390/ijms27104321

**Published:** 2026-05-12

**Authors:** Itzchak Angel, Kalaichitra Periyasamy, Benin Joseph, Erez Aminov

**Affiliations:** 1 Mira Pharmaceuticals, Inc., Miami, FL 33131, USA; 2 Pentagrit Discovery Lab, Chennai 600100, India; kal@pentagrit.com (K.P.); ben@pentagrit.com (B.J.)

**Keywords:** MC4R, obesity, endocannabinoid system, CB1 signaling bias, CB2, MAO-B, zebrafish, reward-associated behavior, nicotine

## Abstract

Obesity resulting from melanocortin-4 receptor (MC4R) dysfunction is characterized by combined metabolic dysregulation and maladaptive reward-related behaviors that limit the durability of existing therapies. The endocannabinoid system is a central regulator of appetite, lipid metabolism, and reward processing; however, first-generation cannabinoid receptor 1 (CB1) antagonists were limited by adverse neuropsychiatric effects. SKNY-1 is an orally active tetrahydrocannabivarin (THCV) analog designed to engage pathway-biased CB1 signaling, modulate cannabinoid receptor 2 (CB2), and selectively inhibit monoamine oxidase B (MAO-B), with the objective of addressing both metabolic and behavioral components of obesity while minimizing central nervous system liability through biased CB1 signaling, CB2 modulation, and potential complementary MAO-B inhibition. Here, we integrated in vitro pharmacological profiling of SKNY-1 with in vivo evaluation in an adult mc4r(G894C) zebrafish model exhibiting obesity-associated metabolic and reward-related phenotypes. In vitro, SKNY-1 displayed low-potency modulation of CB1 cyclic AMP signaling (EC_50_ ~30 µM) but more potent antagonism of the CB1 β-arrestin pathway (IC_50_ ~6 µM), consistent with differential CB1 pathway modulation. SKNY-1 acted as a CB2 partial agonist (EC_50_ ~0.1 µM), with antagonist activity emerging at higher concentrations, and selectively inhibited MAO-B at low affinity with no activity against MAO-A. In vivo, mc4r(G894C) zebrafish mutants exhibited dyslipidemia, hepatic triglyceride accumulation, altered appetite-regulatory gene expression, increased metabolic rate, and enhanced compulsive high-calorie feeding and nicotine-seeking behaviors. Oral administration of SKNY-1 for six days produced dose-dependent effects. Both doses normalized total cholesterol and low-density lipoprotein levels and reduced hepatic triglycerides toward wild-type values without affecting circulating triglycerides. The higher dose (200 ng per fish per day) induced significant body weight reduction while preserving body density and attenuated reward-associated feeding and nicotine-seeking behaviors. The lower dose (20 ng per fish per day) more effectively normalized the leptin a-to-ghrelin expression ratio. Collectively, these findings demonstrate that SKNY-1 engages integrated endocannabinoid and potential dopaminergic mechanisms to improve metabolic parameters and attenuate maladaptive reward-related behaviors in an MC4R-deficient vertebrate model, supporting its further translational investigation for obesity complicated by compulsive eating and substance-seeking behaviors.

## 1. Introduction

Obesity is a multifactorial disorder characterized by dysregulated energy balance, altered lipid metabolism, and maladaptive central reward processing. Loss-of-function mutations in the melanocortin-4 receptor (MC4R) represent one of the most prevalent monogenic causes of obesity and are associated with hyperphagia, leptin resistance, altered energy expenditure, and heightened susceptibility to compulsive and reward-associated behaviors. These clinical and preclinical observations underscore the close integration of hypothalamic metabolic control circuits with mesolimbic reward pathways in the pathophysiology of obesity [1,2].

The endocannabinoid system is a central regulator of appetite, lipid metabolism, insulin sensitivity, inflammation, and reward processing, acting primarily through cannabinoid receptor 1 (CB1) and cannabinoid receptor 2 (CB2) [3,4]. CB1 signaling within the central nervous system promotes feeding and reward-related behaviors, whereas peripheral CB1 activity contributes to lipogenesis and energy storage. CB2 signaling has emerged as an important modulator of immune responses, inflammation, and metabolic homeostasis. Although pharmacological CB1 antagonism can reduce body weight and improve metabolic parameters, the clinical withdrawal of rimonabant highlighted the limitations of non-selective CB1 blockade due to adverse neuropsychiatric effects [4].

**Figure 1:**
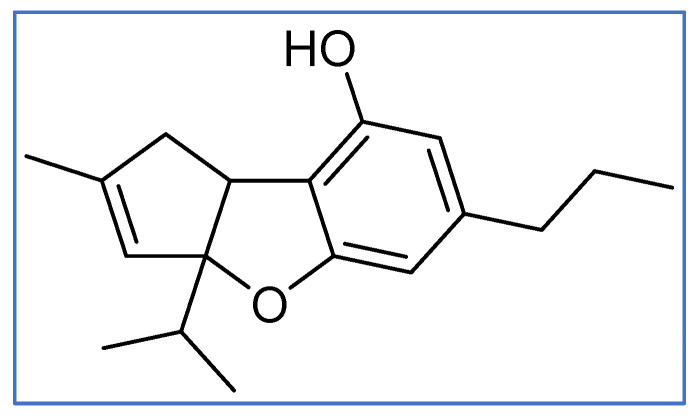
Structure of SKNY-1 (3a-isopropyl-2-methyl-6-propyl-3a,8b-dihydro-1H-cyclopenta[b] benzofuran-8-ol).

These limitations have motivated the development of next-generation cannabinoid-based strategies that emphasize pathway-selective, or biased, CB1 modulation combined with peripheral CB2 engagement and complementary regulation of dopaminergic signaling [5,6]. Δ^9^-Tetrahydrocannabivarin (THCV) is a naturally occurring phytocannabinoid structurally related to Δ^9^-tetrahydrocannabinol (THC) but distinguished by a shortened propyl side chain, resulting in distinct pharmacological properties at cannabinoid receptors. At low doses, THCV is typically non-psychoactive and exhibits a neutral CB1 antagonism profile that differs from classical CB1 inverse agonists, potentially limiting central adverse effects [7,8]. Building on these principles, SKNY-1 is a THCV-derived small molecule designed to combine biased CB1 signaling, CB2 modulation, and selective inhibition of monoamine oxidase B (MAO-B), thereby targeting convergent metabolic and reward-associated pathways implicated in obesity (Figure 1).

Although rodent models remain the gold standard in metabolic research due to their close physiological similarity to humans in adipose tissue biology, thermoregulation, and leptin signaling, the adult mc4r(G894C) zebrafish offers several complementary advantages for initial pharmacological screening. These include high fecundity enabling large sample sizes, low maintenance costs, optical transparency for non-invasive imaging, rapid experimental throughput, and the ability to quantitatively assess integrated metabolic and complex reward-associated behaviors (such as compulsive feeding and nicotine-seeking) in a single vertebrate system. Zebrafish conserve key components of lipid metabolism, appetite regulation, and endocannabinoid signaling relevant to obesity, making them particularly suitable for proof-of-concept evaluation of multi-target compounds before advancing to higher-order mammalian models [9]. Zebrafish also provide a translationally relevant vertebrate model for evaluating integrated metabolic and behavioral pharmacology, as key components of energy balance, lipid metabolism, and reward circuitry are conserved, and complex behaviors such as compulsive feeding and drug-seeking can be quantitatively assessed [9].

In the current therapeutic landscape for obesity, glucagon-like peptide-1 receptor agonists (GLP-1RAs, e.g., semaglutide and tirzepatide) have transformed management by achieving substantial weight loss and improving cardiometabolic outcomes. However, their widespread use is limited by several challenges: high rates of gastrointestinal side effects leading to discontinuation, suboptimal long-term adherence and persistence (often <50% at 1 year in real-world settings), weight regain upon cessation, high cost, and the need for lifelong injectable (or oral) therapy in many patients. These limitations highlight the need for novel oral agents that can address complementary pathways—particularly the maladaptive reward processing and compulsive behaviors that frequently undermine durability of weight loss with existing therapies [10].

In this study, we integrated in vitro characterization of cannabinoid receptor and MAO pharmacology with comprehensive in vivo evaluation of SKNY-1 in an adult mc4r(G894C) zebrafish model exhibiting obesity-associated metabolic and reward-related phenotypes [9,11]. Using metabolic, molecular, neurochemical, and behavioral endpoints, we aimed to determine whether multi-target modulation by SKNY-1 can improve obesity-related abnormalities while maintaining an acceptable tolerability profile.

Loss of melanocortin-4 receptor (MC4R) signaling disrupts hypothalamic regulation of energy balance and alters mesolimbic reward circuitry, contributing to dysregulated appetite control, altered leptin and ghrelin signaling, elevated dopaminergic tone, dyslipidemia, hepatic triglyceride accumulation, and enhanced reward-associated feeding and nicotine-seeking behaviors. SKNY-1 is proposed to engage multiple convergent pathways relevant to these phenotypes. Preferential antagonism of CB1 β-arrestin signaling is hypothesized to modulate central reward-related feeding behaviors without complete CB1 blockade. Partial agonism at CB2 may influence peripheral lipid metabolism and inflammatory signaling, contributing to improvements in circulating cholesterol and hepatic triglyceride levels. Selective inhibition of monoamine oxidase B (MAO-B) is proposed to modulate dopaminergic tone associated with reward-associated behaviors. Collectively, these coordinated central and peripheral actions are proposed to underlie the observed reductions in body weight, improvements in lipid homeostasis, normalization of leptin a-to-ghrl expression balance, and dose-dependent attenuation of reward-associated feeding and nicotine-seeking behaviors in the mc4r(G894C) zebrafish model.

## 2. Results

### 2.1. In Vitro Pharmacological Profile of SKNY-1

**Figure 2:**
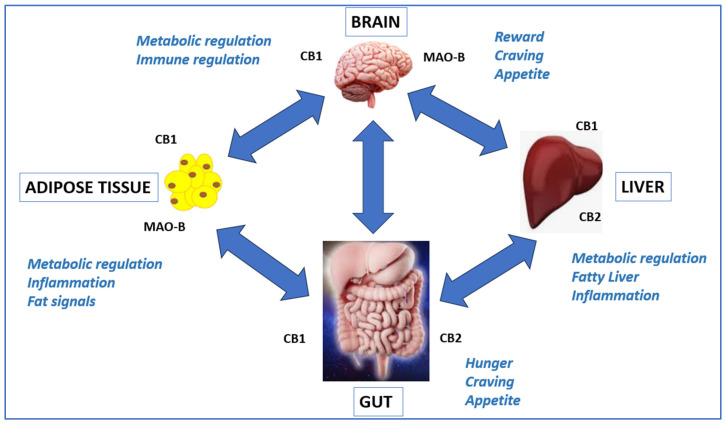
Proposed mechanism of action of SKNY-1.

SKNY-1 exhibited a concentration-dependent, multi-target pharmacological profile across cannabinoid receptor signaling pathways and monoamine oxidase activity (Table 1, Figure 2). At cannabinoid receptor 1 (CB1), SKNY-1 demonstrated low-potency modulation of cyclic AMP signaling, with an EC_50_ of approximately 30 µM, indicating limited engagement of G-protein–mediated CB1 signaling. In contrast, SKNY-1 inhibited CB1 β-arrestin–mediated signaling with greater potency (IC_50_ ~6 µM), revealing differential activity across CB1 signaling pathways.

At cannabinoid receptor 2 (CB2), SKNY-1 acted as a partial agonist with sub-micromolar potency (EC_50_ ~0.1 µM), achieving approximately 60% of maximal receptor activation. At higher concentrations, SKNY-1 exhibited antagonist activity at CB2 (IC_50_ ~30 µM), consistent with a concentration-dependent shift in functional response.

**Figure 3:**
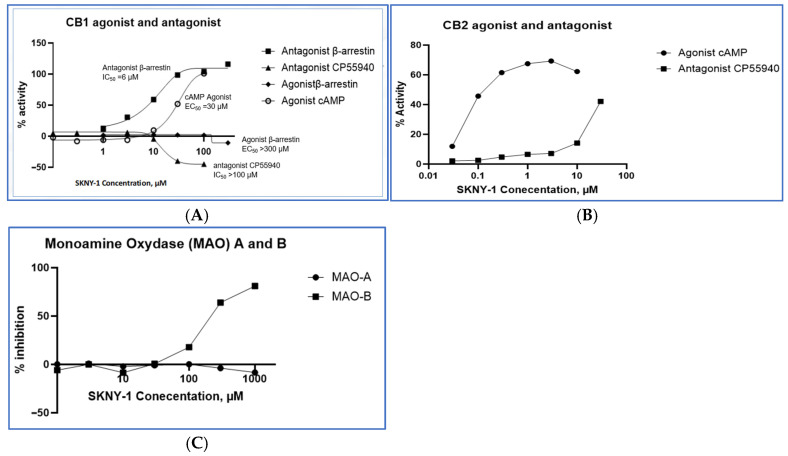
Pharmacological profile of SKNY-1. CB-1 agonist and antagonist activities (**A**), CB-2 agonist and antagonist activities (**B**), and MAO-A and MAO-B inhibition studies (**C**).

In enzymatic assays, SKNY-1 selectively inhibited monoamine oxidase B (MAO-B) with an EC_50_ of approximately 300 µM, while showing minimal inhibition of monoamine oxidase A (MAO-A) at concentrations up to 1000 µM. Together, these data indicate a pharmacological profile characterized by differential engagement of CB1 signaling pathways, dose-dependent modulation of CB2 activity, and relative selectivity for MAO-B over MAO-A. Concentration–response relationships across CB1, CB2, and MAO targets are shown in Figure 3 and summarized in Table 1.

### 2.2. SKNY-1 Reduces Body Weight Without Loss of Whole-Body Density

**Figure 4:**
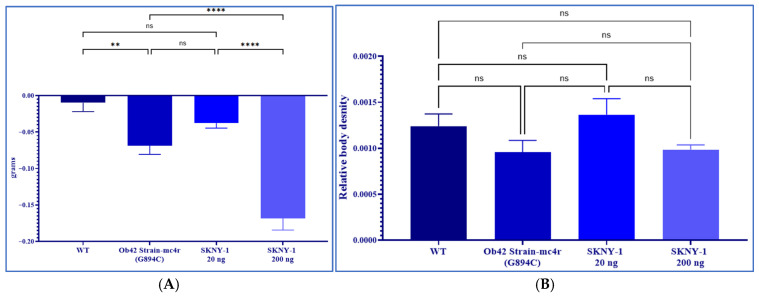
Effects of SKNY-1 on body weight and body density in mc4r(G894C) zebrafish. (**A**) Body weight measurements in wild-type, mc4r(G894C), and SKNY-1–treated zebrafish following six days of oral dosing. SKNY-1 produced a dose-dependent reduction in body weight, with a significant decrease observed at the 200 ng per fish per day dose. (**B**) Relative body density across experimental groups. No significant differences in body density were detected between wild-type, mc4r(G894C), or SKNY-1–treated zebrafish at either dose level. Data are presented as mean ± SD. Statistical significance was assessed by one-way ANOVA with Tukey’s post hoc test; ns, not significant; ** *p* < 0.01; **** *p* < 0.0001.

mc4r(G894C) zebrafish exhibited significantly increased body weight compared with wild-type controls. Oral administration of SKNY-1 resulted in a dose-dependent reduction in body weight, with a pronounced decrease observed at the higher dose (200 ng per fish per day), reaching an average reduction of 30% of baseline body weight. (Figure 4A). The lower dose (20 ng per fish per day) produced a more modest reduction in body weight.

Assessment of whole-body density revealed no significant differences among wild-type, mc4r(G894C), or SKNY-1–treated groups at either dose level (Figure 4B). These findings indicate that SKNY-1–induced body weight reduction occurred without substantial loss of lean tissue over the treatment period.

### 2.3. Effects on Ventilation Rate

**Figure 5:**
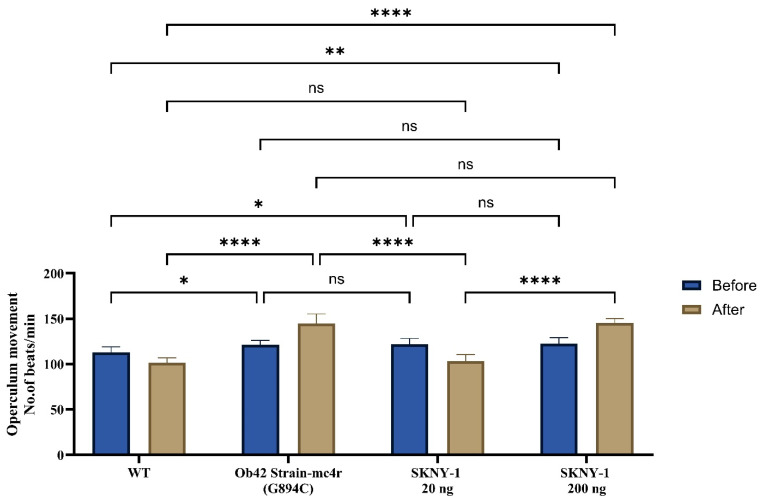
Effects of SKNY-1 on ventilation rate in mc4r(G894C) zebrafish, expressed as opercular movements per minute, in wild-type, mc4r(G894C), and SKNY-1–treated zebrafish following six days of oral dosing. mc4r(G894C) zebrafish exhibited elevated ventilation rates relative to wild-type controls. SKNY-1 treatment modulated ventilation in a dose-dependent manner, with partial normalization observed at the lower dose and sustained elevations observed at the higher dose. Data are presented as mean ± SD. Statistical significance was assessed by one-way ANOVA with Tukey’s post hoc test; ns, not significant; * *p* < 0.05; ** *p* < 0.01; **** *p* < 0.0001.

Baseline ventilation rates were significantly elevated in mc4r(G894C) zebrafish compared with wild-type controls, consistent with an altered metabolic state (Figure 5). SKNY-1 treatment modulated the ventilation rate in a dose-dependent manner. The lower dose (20 ng per fish per day) partially reduced ventilation rates toward wild-type levels, whereas the higher dose (200 ng per fish per day) was associated with sustained elevations in ventilation concurrent with marked body weight reduction.

### 2.4. SKNY-1 Improves Dyslipidemia and Reduces Hepatic Triglyceride Accumulation

**Figure 6:**
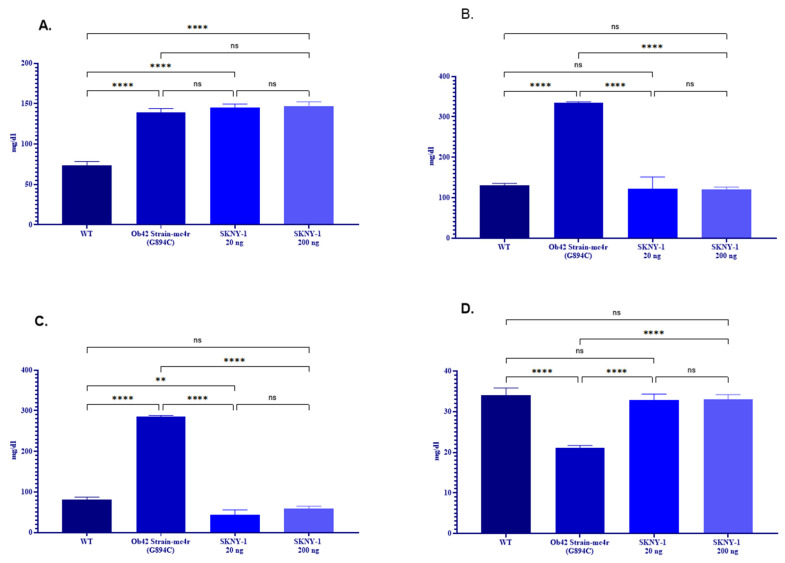
Effects of SKNY-1 on serum lipid profile and hepatic triglyceride content. Serum lipid parameters, including Serum triglycerides (**A**), total cholesterol (**B**), low-density lipoprotein (LDL) (**C**), and high-density lipoprotein (HDL) (**D**), in wild-type, mc4r(G894C), and SKNY-1–treated zebrafish. SKNY-1 treatment normalized total cholesterol and LDL levels and increased HDL levels relative to mc4r(G894C) controls. Data are presented as mean ± SD. Statistical significance was assessed by one-way ANOVA with Tukey’s post hoc test; ns, not significant; ** *p* < 0.01; **** *p* < 0.0001.

mc4r(G894C) zebrafish exhibited pronounced dyslipidemia, characterized by elevated total triglycerides, cholesterol, and low-density lipoprotein (LDL) levels and reduced high-density lipoprotein (HDL) levels relative to wild-type controls (Figure 6A–D). Treatment with SKNY-1 at both dose levels normalized total cholesterol and LDL concentrations toward wild-type values and increased HDL levels. Serum triglyceride levels were not significantly altered by SKNY-1 treatment.

**Figure 7:**
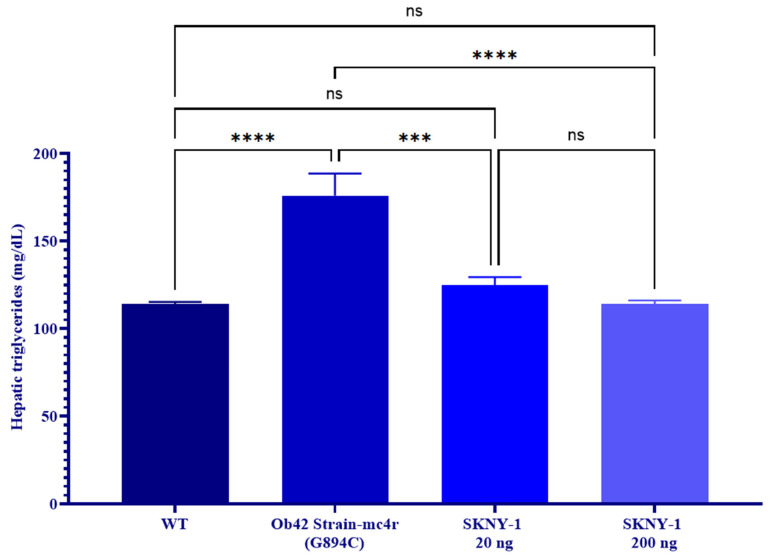
Hepatic triglyceride levels measured in wild-type (WT), mc4r(G894C) (Ob42), and SKNY-1–treated zebrafish following six days of oral dosing. Data are presented as mean ± SD. Statistical analysis was performed using one-way ANOVA followed by Tukey’s post hoc test. The mc4r(G894C) group exhibited significantly elevated hepatic triglyceride levels compared with WT (*p* < 0.0001). SKNY-1 treatment significantly reduced hepatic triglyceride levels at both 20 ng (*p* < 0.001) and 200 ng (*p* < 0.0001) doses relative to mc4r(G894C) controls. No significant differences were observed between WT and either SKNY-1–treated group. ns, not significant; *** *p* <0.001; **** *p* < 0.0001.

In contrast to circulating triglycerides, hepatic triglyceride content was markedly elevated in mc4r(G894C) zebrafish and was significantly reduced following SKNY-1 treatment at both doses, reaching levels comparable to wild-type animals (Figure 7).

### 2.5. Appetite-Regulatory Gene Expression

**Figure 8:**
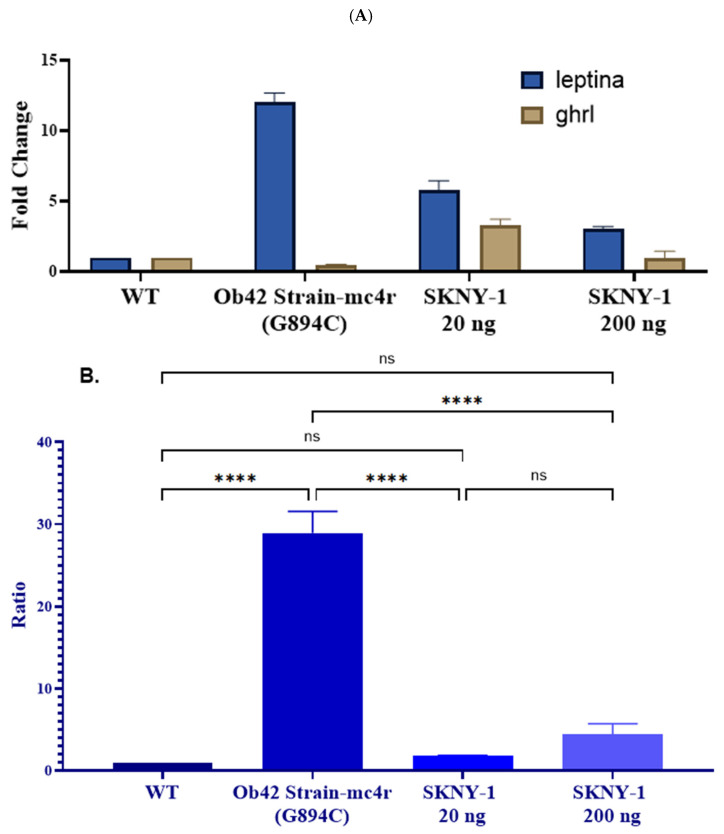
Effects of SKNY-1 on appetite-regulatory gene expression in mc4r(G894C) zebrafish. (**A**) Relative expression levels of *leptin a* and *ghrl* measured by RT-PCR in wild-type, mc4r(G894C), and SKNY-1–treated zebrafish following six days of oral dosing. mc4r(G894C) zebrafish exhibited increased *leptin a* expression and reduced *ghrl* expression relative to wild-type controls. (**B**) Ratio of *leptin a*-to-*ghrl* expression across experimental groups. SKNY-1 treatment significantly reduced the elevated *leptin a*-to-*ghrl* ratio observed in mc4r(G894C) zebrafish, with the greatest normalization observed at the lower dose. Data are presented as mean ± SD. Statistical significance was assessed by one-way ANOVA with Tukey’s post hoc test; ns, not significant; **** *p* < 0.0001.

mc4r(G894C) zebrafish exhibited marked dysregulation of appetite-regulatory gene expression, characterized by elevated *leptin a* expression and reduced *ghrl* expression relative to wild-type controls (Figure 8A). This resulted in a significantly increased *leptin a*-to-*ghrl* expression ratio in the mc4r(G894C) model (Figure 8B).

Treatment with SKNY-1 shifted the expression of both genes toward wild-type patterns in a dose-dependent manner. The lower dose (20 ng per fish per day) produced the most pronounced normalization of the *leptin a*-to-*ghrl* ratio, while the higher dose (200 ng per fish per day) also significantly reduced the ratio relative to mc4r(G894C) controls, although to a lesser extent (Figure 8B).

### 2.6. Suppression of Compulsive Feeding and Nicotine-Seeking Behaviors

**Figure 9:**
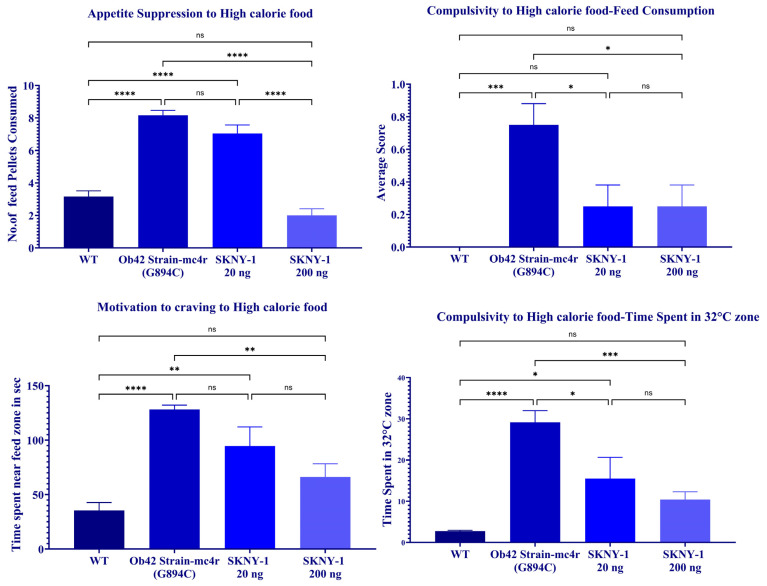

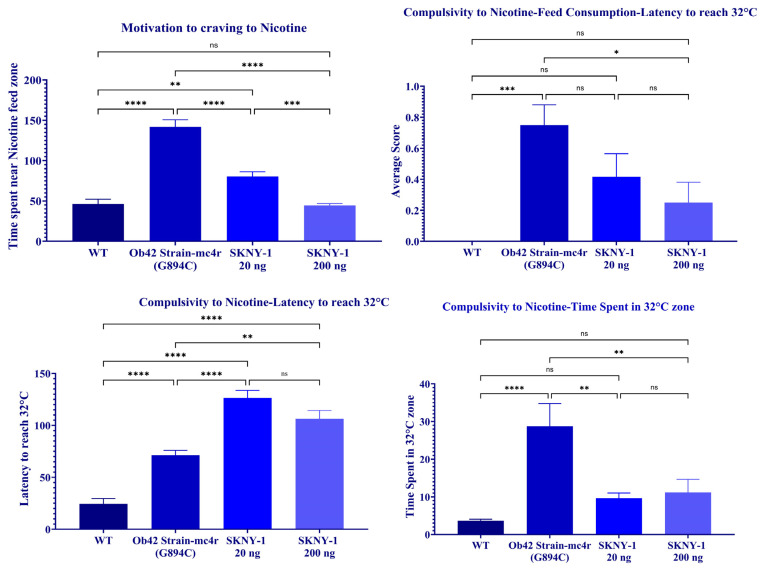
Effects of SKNY-1 on feeding- and nicotine-associated behaviors in mc4r(G894C) zebrafish. Behavioral assessments of feeding- and nicotine-associated behaviors in wild-type, mc4r(G894C), and SKNY-1–treated zebrafish following six days of oral dosing. Panels depict measures of high-calorie food consumption, persistence in aversive zones during food access, latency to enter reward-associated zones, and time spent in reward-associated zones for both food- and nicotine-related paradigms. mc4r(G894C) zebrafish exhibited increased food consumption, enhanced aversive-zone persistence, reduced latency to enter reward-associated zones, and increased nicotine-seeking behavior relative to wild-type controls. SKNY-1 treatment produced dose-dependent attenuation of these behaviors, with partial effects observed at 20 ng per fish per day and more pronounced suppression at 200 ng per fish per day. Data are presented as mean ± SD. Statistical significance was assessed by one-way ANOVA with Tukey’s post hoc test; ns, not significant; * *p* < 0.05; ** *p* < 0.01; *** *p* < 0.001; **** *p* < 0.0001.

mc4r(G894C) zebrafish exhibited marked alterations in feeding and nicotine-related behaviors compared with wild-type controls, including increased consumption of high-calorie food, greater persistence in aversive zones during food access, and enhanced nicotine-seeking behavior across multiple behavioral paradigms (Figure 9).

Treatment with SKNY-1 attenuated these behaviors in a dose-dependent manner. The lower dose (20 ng per fish per day) produced partial reductions in high-calorie food consumption, aversive-zone persistence, and nicotine-seeking metrics. In contrast, the higher dose (200 ng per fish per day) produced robust suppression across behavioral endpoints, including reduced pellet consumption, decreased time spent in aversive zones, increased latency to enter reward-associated zones, and diminished nicotine-seeking behavior. For multiple parameters, behavioral responses in the high-dose group were not significantly different from those observed in wild-type zebrafish (Figure 9).

## 3. Discussion

In this study, we combined in vitro pharmacological characterization with in vivo metabolic, molecular, neurochemical, and behavioral analyses to evaluate SKNY-1, a THCV-derived small molecule designed to engage multiple pathways relevant to obesity and reward-associated behaviors. Using an adult mc4r(G894C) zebrafish model, we show that SKNY-1 produces dose-dependent improvements in body weight, lipid homeostasis, hepatic triglyceride accumulation, appetite-regulatory gene expression, and feeding- and nicotine-associated behaviors, while exhibiting differential tolerability across doses.

At the receptor level, SKNY-1 displayed differential engagement of CB1 signaling pathways, with relatively low potency in G-protein–mediated cyclic AMP signaling and greater potency in inhibiting β-arrestin–mediated signaling. Although formal bias quantification was not performed, this pattern of pathway engagement is consistent with differential CB1 pathway modulation and contrasts with non-selective CB1 antagonism, which has been associated with adverse neuropsychiatric effects in clinical settings [5,6,12,13]. In parallel, SKNY-1 acted as a partial agonist at CB2 at sub-micromolar concentrations, with antagonist activity emerging at higher concentrations, suggesting concentration-dependent modulation of peripheral cannabinoid signaling. SKNY-1 also exhibited relative selectivity for MAO-B over MAO-A, providing a potential additional mechanism by which dopaminergic tone may be modulated without broad monoamine disruption. While SKNY-1 demonstrated selectivity for MAO-B over MAO-A, the observed MAO-B inhibitory potency was low (EC_50_ ~300 µM). In the absence of exposure and brain/plasma concentration data, MAO-B inhibition should be considered a potential ancillary mechanism or a possible contributing factor to dopaminergic tone regulation and reward-behavior attenuation, but emphasize that the primary observed effects are more plausibly linked to the cannabinoid receptor activities (biased CB1 and CB2 partial agonism). Future studies will include PK/exposure–response analysis to determine whether MAO-B engagement occurs at pharmacologically relevant concentrations.

In vivo, these pharmacological properties were associated with meaningful metabolic effects in the mc4r(G894C) model. SKNY-1 reduced body weight without detectable loss of body density. Given that skeletal muscle represents the largest single soft-tissue mass compartment (~35–60% of body weight), the stability of whole-body density supports that the observed weight loss was not accompanied by substantial loss of lean tissue (primarily muscle). These changes were accompanied by improved dyslipidemia, including normalization of total cholesterol and LDL levels and marked reduction in hepatic triglyceride accumulation. Notably, serum triglyceride levels were not significantly altered, highlighting a dissociation between circulating and hepatic lipid handling. Ventilation rate, used here as a surrogate marker of metabolic state, was differentially affected by dose, with partial normalization at the lower dose and sustained elevation at the higher dose, coincident with greater weight loss.

SKNY-1 also modulated appetite-regulatory gene expression, reducing the elevated *leptin a*-to-*ghrl* expression ratio observed in mc4r(G894C) zebrafish. This molecular profile was accompanied by dose-dependent attenuation of feeding- and nicotine-associated behaviors across multiple behavioral paradigms. The higher dose produced robust attenuation of high-calorie food consumption, aversive-zone persistence, and nicotine-seeking behaviors, with several measures returning to levels not significantly different from wild-type controls.

The observed changes in appetite-regulatory gene expression merit careful interpretation. In the mc4r(G894C) zebrafish, the marked increase in leptin a (lepa) expression and the corresponding elevation in the leptin a-to-ghrelin (ghrl) ratio are most likely secondary to the increased adiposity and positive energy balance characteristic of MC4R deficiency, rather than a primary direct dysregulation of central appetite control circuits. In both mammalian and zebrafish models of obesity, elevated adipose mass drives higher leptin production as a physiological feedback signal; however, downstream MC4R signaling deficiency renders the animals centrally leptin-resistant, such that the elevated leptin fails to effectively suppress feeding [9,14,15]. Similarly, the reduced ghrelin expression observed in the mutant line is consistent with suppression secondary to chronic positive energy balance and expanded fat stores, as ghrelin levels are typically downregulated in states of obesity and overnutrition.

Treatment with SKNY-1 shifted lepa and ghrl expression patterns toward wild-type levels in a dose-dependent manner, with the lower dose producing the most effective normalization of the leptin a-to-ghrelin ratio. These molecular improvements occurred alongside reductions in body weight, hepatic triglycerides, and dyslipidemia, suggesting that SKNY-1 may indirectly restore aspects of energy balance sensing, possibly through its multi-target actions on cannabinoid signaling and also eventually on dopaminergic tone. Nevertheless, because the gene expression changes appear largely secondary to alterations in adiposity, they should be viewed as supportive biomarkers of improved metabolic status rather than as primary evidence of direct hypothalamic appetite regulation by SKNY-1. Future studies incorporating measurements of circulating leptin and ghrelin protein levels, as well as central leptin sensitivity assays, would help further clarify the mechanistic relationship.

An important finding of this study is the apparent dissociation between dose-dependent efficacy and tolerability. The lower dose of SKNY-1 preferentially normalized neurochemical and gene expression markers and was well tolerated, whereas the higher dose produced greater effects on body weight and behavior. This pattern suggests concentration-dependent engagement of SKNY-1’s multiple targets and underscores the importance of dose optimization when pursuing multi-target pharmacological strategies. These data suggest a dose range in which metabolic and behavioral benefits can be balanced against tolerability considerations.

Several limitations should be acknowledged. The treatment duration was relatively short, and systemic exposure, pharmacokinetics, and receptor occupancy were not assessed. Additionally, while zebrafish provide a powerful and translationally relevant vertebrate model, extrapolation to mammalian systems will require confirmation in higher-order models. This timeframe was intentionally selected as an initial proof-of-concept design to rapidly evaluate the pharmacological activity of the compound across multiple integrated metabolic, molecular, and behavioral endpoints in the adult mc4r(G894C) zebrafish model, while minimizing the risk of cumulative toxicity, receptor adaptation, or behavioral habituation that can occur with prolonged exposure in small vertebrate models. Nevertheless, this short course does not permit assessment of the durability or sustainability of the observed improvements in body weight, lipid homeostasis (including normalization of cholesterol and LDL levels and reduction in hepatic triglycerides), appetite-regulatory gene expression, or—most critically—the attenuation of compulsive feeding and nicotine-seeking behaviors. The behavioral effects, in particular, may include a significant acute pharmacological component rather than reflecting long-term modulation of reward circuitry, and it remains unknown whether the metabolic benefits would be maintained, diminish, or even rebound following treatment cessation.

In zebrafish pharmacological research, short-term dosing regimens (typically ranging from several days to 1–2 weeks) are commonly employed for initial screening of metabolic and neurobehavioral agents, as they allow efficient evaluation of on-target activity in a high-throughput vertebrate system. Longer-term studies (often spanning weeks to months) are more typical for establishing diet-induced obesity phenotypes themselves or for evaluating chronic efficacy and safety in mammalian models [16]. Consequently, the current findings should be interpreted as demonstrating promising acute-to-subacute effects that warrant further investigation rather than as evidence of sustained therapeutic benefit. Extended dosing paradigms, including washout periods and longitudinal monitoring of body weight, lipid parameters, and reward-associated behaviors, will be essential in future studies to determine the durability of SKNY-1’s actions and to better model the chronic nature of human obesity and associated compulsive behaviors. Such studies will also enable a more comprehensive evaluation of long-term safety and tolerability.

In summary, this work indicates that SKNY-1 engages convergent central and peripheral pathways implicated in obesity and reward-associated behaviors in an MC4R-deficient model. By integrating differential CB1 pathway engagement, concentration-dependent CB2 modulation, and relative MAO-B selectivity, SKNY-1 represents a pharmacological approach that may address both metabolic dysregulation and maladaptive reward-driven behaviors. These findings support further investigation of SKNY-1 and related compounds as potential candidates for multifactorial metabolic disorders.

## 4. Materials and Methods

### 4.1. Materials

SKNY-1 (3a-isopropyl-2-methyl-6-propyl-3a,8b-dihydro-1H-cyclopenta[b] benzofuran-8-ol) was synthetized by Scinai Biopharma Services Ltd. (Yavne, Israel) to a purity of >95%. All the chemicals purchased for the study were of analytical grade. Chemicals used in the study are as follows: Tricaine (T0941), Agarose (SRL 9012-36-6), Nicotine (Sigma-Aldrich, St. Louis, MO, USA, CAS No. 22083-74-5), Pro TechEx—Serum Triglycerides (Chennai, India) (Cat. No: OP-3721-10xp), Pro TechEx—VLDL Cholesterol kit (Cat. No: OP-3717V-10xp), Pro TechEx—LDL Cholesterol Kit (Cat. No: OP-3717L-10xp), Pro TechEx—Blood Cholesterol Estimation by CHOD-POD kit (Cat. No: OP-3717-20xp), Pro TechEx—HDL Cholesterol kit (Cat. No: OP-3717H-10xp).

### 4.2. In Vitro Pharmacology: CB1 and CB2 Activities

CB1 agonist binding was performed using human recombinant (Chem-RBL) cells and [^3^H]CP 55940 as the ligand, according to the method described in [17].

CB1 and CB2 agonist activity was evaluated in human recombinant (CHO) cells using HTRF-based cAMP assays as the measured component [18]. For the evaluation of CB1 antagonist activity, CP 55940 (1 nM) was used as the reference agonist, and either cAMP or β-arrestin was used as the readout [18]. For CB2 antagonist activity, WIN 55212-2 (3 nM) was used as the reference ligand [18].

### 4.3. In Vitro Pharmacology: MAO-A and MAO-B Activities

Enzymatic MAO-A and MAO-B activities were evaluated using the respective human recombinant enzymes. For MAO-B, the assay was conducted using a D-luciferin derivative (4 µM) as the substrate, with luminescence of methyl-ester luciferin measured as the readout. For MAO-A, kynuramine dihydrochloride (350 µM) was used as the substrate, and the formation of 4-hydroxyquinoline was measured spectrophotometrically [19].

### 4.4. Model Induction

The forward genetic method, as per Solnica-Kreze et al., 1993 [20], was employed to develop mutant lines. Adult zebrafish were subjected to the ENU chemical mutagen to induce random mutagenesis, administered through the water dissolution method. Prior to the spawning, the ENU-induced male zebrafish and adult females were housed separately under standard laboratory husbandry conditions. Zebrafish founders were set for spawning at a spawning ratio of female to male fish of 1:4 per breeding tank. The F1 progeny were screened, and the resulting mutants (G891C) were inbred to generate stable lines. The nomenclature for the model was decided to be G894C since it is an ortholog from Humans for that particular site. For the current study, stable heterozygous founders were inbred to generate homozygous mutants with the genotype Gene ID: 286833, Gene-mc4r [melanocortin 4 receptor] that was used for the study. The mutant embryos were housed and maintained in embryo medium. Quality check of the embryonic development was completed using Labomed LX400 brightfield microscope (Castaic, CA, USA) with Labomed Camera LC-5 1080P C-MOUNT WIFI CMOS (Los Angeles, CA, USA). The embryos displaying an opaque discoloration were repudiated, and only the embryos in the best growth phase were selected for the study. The selected embryos were transferred to a two-liter housing tank filled with water and were housed in a ratio of 80 per housing tank of 25 L capacity, thereby providing adequate space for swimming motion and minimizing the factor of crowding. Screening for mutants was carried out on 12 dpf for the manifestation of behavioral phenotype with increased feeding behavior and restricted movement, and the selected larvae were advanced for the study. During the larval to adult developmental stages (0 dpf to 180 dpf), the study tanks were conditioned (under a water temperature of 27 ± 1 °C and pH between 7.2 and 7.4).

### 4.5. Zebrafish Husbandry and Ethics

Wild-type and mc4r(G894C) zebrafish (*Danio rerio*) were maintained under standardized laboratory conditions, including a water temperature of 27 ± 1 °C, pH 7.2–7.4, and a 14 h light/10 h dark photoperiod. Fish were housed and handled in accordance with established zebrafish husbandry guidelines.

All experimental procedures were conducted in an AAALAC-accredited facility and were reviewed and approved by the Institutional Animal Ethics Committee (Authorization PNT016 from 19 August 2025). Animal care and experimental protocols complied with the guidelines of the Committee for the Purpose of Control and Supervision of Experiments on Animals (CPCSEA) and adhered to internationally accepted standards for the ethical use of vertebrate animals in research.

### 4.6. Ventilation Rate

Adult zebrafish ventilation rate was assessed post-habituation phase (pre-treatment) and post-treatment. Ventilation rate was quantified by counting opercular movements (gill cover beats) over a 1 min period using direct visual observation under controlled conditions. Fish were placed individually in double-walled observation chambers containing housing water at consistent temperature and lighting. A 5 min acclimation period was provided before recording to minimize handling-related adaptation. Measurements were taken at the same time of day, post-habituation and post-treatment phases, to reduce circadian variability. Data were expressed as opercular beats per minute (bpm), and changes in ventilation rate were compared across time points and experimental groups to evaluate the physiological effects of treatment.

### 4.7. mc4r(G894C) Obesity and Reward-Related Behavioral Model

The Ob42 mc4r(G894C) zebrafish line was generated by N-ethyl-N-nitrosourea (ENU)–based forward mutagenesis and bred to homozygosity. This line exhibits reproducible obesity-associated metabolic abnormalities and alterations in reward-related behaviors in adulthood. Prior to treatment, adult fish were habituated to a high-calorie diet and to nicotine exposure using established conditioning paradigms to establish consistent feeding motivation and nicotine-seeking behaviors. These procedures were used to generate a reproducible model of obesity accompanied by maladaptive reward-related behavioral phenotypes.

### 4.8. Study Design and Dosing

Adult zebrafish were randomly assigned to one of four experimental groups (n = 60 per group) within each genetic background: wild-type control and then mc4r(G894C) model control, SKNY-1 low-dose treatment (20 ng per fish per day), or SKNY-1 high-dose treatment (200 ng per fish per day). SKNY-1 was administered orally via compound-infused food pellets once daily for six consecutive days. Endpoint measurements were conducted on day 7 following completion of the dosing period. All dosing and assessments were performed in a blinded manner where feasible. A six-day oral dosing regimen was selected as an initial proof-of-concept to evaluate rapid pharmacological activity while limiting potential adaptation or off-target effects in this small vertebrate model.

**Figure 10:**
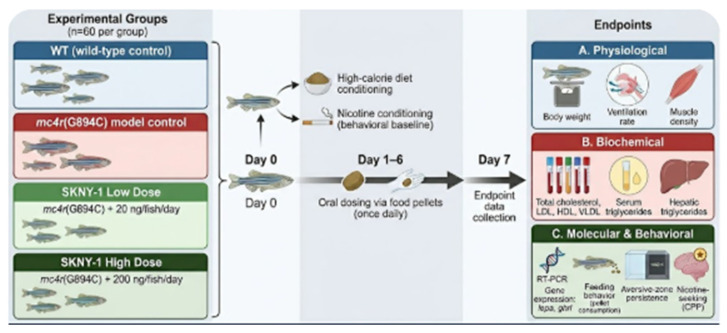
Schematic design of the study.

The design of the study is summarized in the figure below (Figure 10):

### 4.9. Physiological and Biochemical Endpoints

Physiological endpoints included body weight, ventilation rate (quantified as opercular movements per minute), and body density. Biochemical assessments included serum lipid profiling, comprising total cholesterol, low-density lipoprotein (LDL), high-density lipoprotein (HDL), very-low-density lipoprotein (VLDL), and triglycerides, as well as hepatic triglyceride content.

### 4.10. Gene Expression Analysis

To measure the gene expression, adult zebrafish (N = 6) from respective groups was sedated using Tricaine just before the sample collection and a small portion of the caudal fin was amputated for DNA amplification.

Total RNA was isolated from zebrafish tissue samples using standard extraction methods. Complementary DNA was synthesized by reverse transcription, and gene expression levels of leptin a (lepa) and *ghrelin* (ghrl) were quantified by reverse transcription–polymerase chain reaction (RT-PCR) (Table 2). *β*-actin was used as the internal reference gene for normalization. Relative gene expression was calculated using the comparative threshold cycle (2^−ΔΔCt^) method, and the ratio of *leptin a*-to-*ghrl* expression was derived for each experimental group.

### 4.11. Behavioral Assays

Behavioral assessments were conducted to evaluate feeding motivation and reward-associated behaviors as described below:

#### 4.11.1. Appetite Suppression and Compulsive Feeding

The appetite suppression assay in adult zebrafish is designed to evaluate the effects of test compounds on feeding behavior. Prior to the assay, the fish were acclimated to experimental tanks and feeding schedules. On the day of the assay, the fish were fasted for 24 h to ensure a uniform hunger state. Following fasting, fish were placed individually and administered the test compound via oral feed. After a predetermined absorption period (e.g., 30 min to 1 h), a known quantity of standardized commercial feed was introduced. Feeding behavior was monitored for a fixed time window, usually 5–10 min, and the number of pellets consumed was documented and analyzed. Reduced food intake or increased latency relative to controls was interpreted as evidence of appetite suppression. All observations were performed under consistent lighting and environmental conditions.

#### 4.11.2. Compulsivity to High-Calorie Food

To assess compulsive food-seeking behavior during aversive consequences, adult zebrafish were subjected to a modified feeding environment. To perform the assay, the fish was transferred from the housing environment, 27 +/− 1 °C, to a temperature-regulated tank with two zones: 27 +/− 1 °C and 32 +/− 1 °C. The change in temperature induces a challenge to the fish to maintain body physiology at optimum. During this timeline, the high-calorie feed pellet is placed in the water maintained at an elevated temperature of 32 °C, serving as a mild aversive stimulus. Fish behavior was recorded over a fixed observation period of 5 min, during which time latency to enter the aversive zone, duration spent in the aversive zone, and successful food retrieval were quantified. Increased willingness to enter and feed in the aversive zone despite the thermal discomfort was interpreted as a measure of compulsive food-seeking or addiction-like behavior. The control and treatment groups were tested under identical conditions.

#### 4.11.3. Motivation to Craving High-Calorie Food

To assess craving-related motivated behavior, adult zebrafish were subjected to a conditioned place preference using a Y-shaped tank apparatus, which enables clear spatial differentiation between cues. Each arm of the Y-tank was distinctly marked using visual cues, such as contrasting colored panels, red and green, allowing for cue-reward pairing. The test was comprised of a conditioning and a test phase. During the conditioning phase, over 3 days before the test, individual fish were confined to one visually distinct arm of the Y-tank for a fixed duration of 5 min each day, where they received a rewarding stimulus, a high-calorie feed. The opposite arm served as a non-reward-paired control zone. The central arm remained neutral and unmarked. 

After conditioning, a test session was conducted in which zebrafish were allowed free access to all three arms of the Y-tank for a defined observation period of 5 min. Behavioral endpoints, time spent in each arm, were recorded using overhead video tracking. Persistence in exploring or occupying the reward-paired arm in the absence of the reward is interpreted as a measure of craving or motivated seeking behavior.

#### 4.11.4. Compulsivity to Nicotine

To assess compulsive food-seeking behavior during aversive consequences, adult zebrafish were subjected to a modified feeding environment. To perform the assay, the fish were transferred from the housing environment 27 +/− 1 °C to a temperature-regulated tank with two zones: 27 +/− 1 °C and 32 +/− 1 °C. The change in temperature induces a challenge to the fish to maintain body physiology at optimum. During this timeline, the nicotine-infused pellet is placed in the water maintained at an elevated temperature of 32 °C, serving as a mild aversive stimulus. Fish behavior was recorded over a fixed observation period of 5 min, during which time latency to enter the aversive zone, duration spent in the aversive zone, and successful nicotine feed retrieval were quantified. Increased willingness to enter and feed in the aversive zone despite the thermal discomfort was interpreted as a measure of compulsive food-seeking or addiction-like behavior. Control and treatment groups were tested under identical conditions.

#### 4.11.5. Motivation to Craving to Nicotine

To assess craving-related motivated behavior, adult zebrafish were subjected to a conditioned place preference using a Y-shaped tank apparatus, which enables clear spatial differentiation between cues. Each arm of the Y-tank was distinctly marked using visual cues, such as contrasting colored panels, red and green, allowing for cue-reward pairing. The test was comprised of a conditioning and a test phase. During the conditioning phase over 3 days before the test, individual fish were confined to one visually distinct arm of the Y-tank for a fixed duration of 5 min each day, where they received a rewarding stimulus, a nicotine-infused feed. The opposite arm served as a non-reward-paired control zone. The central arm remained neutral and unmarked. 

After conditioning, a test session was conducted in which zebrafish were allowed free access to all three arms of the Y-tank for a defined observation period of 5 min. Behavioral endpoints, time spent in each arm, were recorded using overhead video tracking. Persistence in exploring or occupying the reward-paired arm in the absence of the reward is interpreted as a measure of craving or motivated seeking behavior.

### 4.12. Body Weight

Adult zebrafish body weight was measured individually after habituation (before treatment) and after the treatment period to assess weight changes associated with the intervention. Prior to weighing, fish were fasted for a minimum of 12 h to reduce variability due to gut content. Each fish was gently transferred, briefly blotted on a sponge to remove excess water, and placed on a pre-tared analytical balance with ±0.001 g sensitivity to record baseline weight. The same procedure was repeated at the end of the treatment period under identical fasting and handling conditions. Body weight change for each fish was calculated as the difference between post-treatment and pre-treatment weights and expressed as a percentage change relative to baseline. All weighing was conducted at the same time of day to minimize variation, with consistent environmental conditions maintained throughout the study.

Change in body weight and percentage change in body weight normalized to the control were calculated.

Change in Body Weight = Post-treatment weight − Pre-treatment weight

Percentage Change = {Post Treatment/Pre-Treatment weight} × 100

### 4.13. Whole-Body Density

Body mass quantification employs hydrodensitometry to accurately measure body density by evaluating water displacement with the known volume of water. The difference in initial and final water level is documented and used to calculate the volume of water displaced. Body density is calculated by dividing the mass on land (g) by the volume of water displaced (cm)^3^.

Body density = Mass on land (g)/Volume of water displaced

### 4.14. Statistical Analysis

Data are presented as mean ± standard deviation (SD). Statistical analyses were performed using one-way analysis of variance (ANOVA) for comparisons among multiple groups, followed by Tukey’s post hoc test where appropriate. A two-sided *p*-value < 0.05 was considered statistically significant.

## 5. Conclusions

Short-term treatment with SKNY-1 produced coordinated improvements in metabolic, molecular, and behavioral endpoints in an mc4r(G894C) zebrafish model of obesity-associated dysregulation. These effects were observed alongside a pharmacological profile characterized by differential engagement of CB1 signaling pathways, concentration-dependent modulation of CB2 activity, and relative selectivity for MAO-B. Together, the findings provide a rationale for further investigation of SKNY-1 as a multi-target pharmacological approach for obesity and related reward-associated behaviors.

## Figures and Tables

**Table 1:** In Vitro Pharmacological Profile of SKNY-1.

Target	Assay/Pathway	Activity	Potency (Approx.)	Interpretation
CB1	agonist binding	Low-potency (Cmax = 100%)	IC_50_ = 47 µM	
CB1	cAMP signaling	Low-potency modulation (Cmax = 100%)	EC_50_ ~30 µM	Limited engagement of CB1 G-protein–mediated signaling
CB1	β-arrestin pathway	Antagonist (Cmax = 100%)	IC_50_ ~6 µM	Preferential inhibition of CB1 β-arrestin signaling, consistent with pathway-selective modulation
CB2	Functional response	Partial agonist (Emax ~60%)	EC_50_ ~0.1 µM	Partial activation of CB2 signaling at sub-micromolar concentrations
CB2	Functional response (higher concentrations)	Antagonist (Cmax = 50%@30 µM)	IC_50_ ~30 µM	Concentration-dependent transition to antagonistic activity
MAO-B	Enzymatic inhibition	Low-potency Inhibitor (Cmax = 100%)	EC_50_ ~300 µM	Selective inhibition of MAO-B enzymatic activity
MAO-A	Enzymatic inhibition	Minimal activity	EC_50_ > 1000 µM	Limited inhibition of MAO-A relative to MAO-B

**Table 2:** Primer sequences used to quantify gene expression by real-time PCR.

Gene Mix	Primer Sequence Forward	Primer Sequence Reverse	Product Length
*Leptin a*	CCAGAGATTCCCGCTGACAA	CTCGGCGTATCTGGTCAACA	118 bp
*Ghrl*	GACTCAGAAACCGCAGGGTC	CAATGCACCCACTTTGCTACAG	361 bp
*Beta-actin*	GCATTGCTGACCGTATGCAG	GAAGCACTTCCTGTGGACGA	203 bp

## Data Availability

The original contributions presented in this study are included in the article. Further inquiries can be directed to the corresponding author.

## Abbreviations

AAALAC	Association for Assessment and Accreditation of Laboratory Animal Care
ANOVA	analysis of variance
cAMP	cyclic adenosine monophosphate
CB1	cannabinoid receptor type 1
CB2	cannabinoid receptor type 2
CNS	central nervous system
CPCSEA	Committee for the Purpose of Control and Supervision of Experiments on Animals
EC_50_	half maximal effective concentration
ELISA	enzyme-linked immunosorbent assay
ENU	N-ethyl-N-nitrosourea
ghrl	ghrelin
HDL	high-density lipoprotein
IC_50_	half maximal inhibitory concentration
LDL	low-density lipoprotein
MAO-A	monoamine oxidase A
MAO-B	monoamine oxidase B
MC4R	melanocortin-4 receptor
RT-PCR	reverse transcription polymerase chain reaction
SD	standard deviation
THC	Δ^9^-tetrahydrocannabinol
THCV	Δ^9^-tetrahydrocannabivarin
VLDL	very-low-density lipoprotein
WT	wild-type
